# Giant Cerebral Aneurysm Rupture in an Ischemic Stroke

**DOI:** 10.31662/jmaj.2022-0029

**Published:** 2022-05-30

**Authors:** Tatsuya Tanaka

**Affiliations:** 1 Department of Neurosurgery, International University of Health and Welfare, School of Medicine, Narita, Japan

**Keywords:** ischemic stroke, subarachnoid hemorrhage, giant aneurysm, imminent rupture

A 68-year-old man presented with a sudden onset of right hemiparesis and aphasia. Computed tomography (CT) revealed a high-density, round mass in the left middle cerebral artery (MCA) suspected to be an unruptured giant cerebral aneurysm (Figure 1A). CT angiography revealed MCA occlusion and an aneurysm in the proximal portion of the left MCA with poor opacification, suggesting partial thrombosis (Figure 1B and 1C). The patient was diagnosed with cerebral infarction due to an embolism from a partially thrombosed aneurysm or thrombotic occlusion of the distal MCA; antithrombotic therapy was started. On day 3, he suddenly became comatose. CT indicated subarachnoid hemorrhage (SAH) and cerebral infarction in the territory of the MCA (Figure 2A and B). Giant intracranial aneurysms, having diameter >25 mm, represent approximately 5% of intracranial aneurysms ^(1)^. Cerebral infarction due to aneurysm thrombosis in giant intracranial aneurysms is rare ^(1)^. Patients with ischemic stroke distal to an unruptured intracranial aneurysm often experience SAH after the stroke and have extremely poor prognosis ^(2)^.

**Figure 1.**
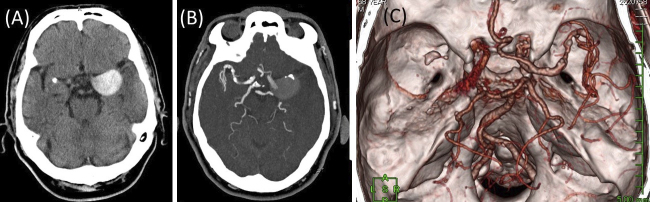
Computed tomography (CT) on admission revealed a high-density, round mass in the left middle cerebral artery (MCA), which was suspected to be an unruptured giant cerebral aneurysm (A). CT angiography revealed MCA occlusion and an aneurysm in the proximal part of the left MCA with poor opacification, suggesting partial thrombosis (B, C).

**Figure 2.**
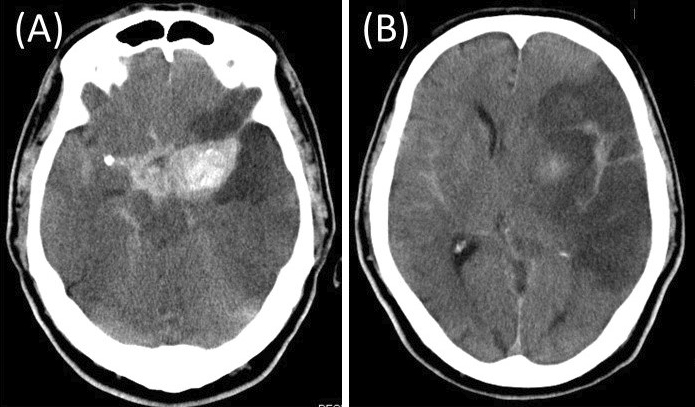
Computed tomography indicating a subarachnoid hemorrhage (A) and cerebral infarction in the territory of the MCA (B).

## Article Information

### Conflicts of Interest

None

### Author Contributions

TT wrote the first draft and managed all of the submission processes.

### Informed Consent

We have obtained informed consent for this case report.
